# Effect of Caloric Restriction on the *in vivo* Functional Properties of Aging Microglia

**DOI:** 10.3389/fimmu.2020.00750

**Published:** 2020-04-28

**Authors:** Maria Olmedillas del Moral, Nicole Fröhlich, Katherine Figarella, Nima Mojtahedi, Olga Garaschuk

**Affiliations:** Department of Neurophysiology, Institute of Physiology, Eberhard Karls University Tübingen, Tübingen, Germany

**Keywords:** caloric restriction, microglia, aging, sex differences, *in vivo* Cacpsdummy2+ imaging, process motility

## Abstract

Throughout the lifespan, microglia, the primary innate immune cells of the brain, fulfill a plethora of homeostatic as well as active immune defense functions, and their aging-induced dysfunctionality is now considered as a key trigger of aging-related brain disorders. Recent evidence suggests that both organism’s sex and age critically impact the functional state of microglia but *in vivo* determinants of such state(s) remain unclear. Therefore, we analyzed *in vivo* the sex-specific functional states of microglia in young adult, middle aged and old wild type mice by means of multicolor two-photon imaging, using the microglial Ca^2 +^ signaling and directed process motility as main readouts. Our data revealed the sex-specific differences in microglial Ca^2 +^ signaling at all ages tested, beginning with young adults. Furthermore, for both sexes it showed that during the lifespan the functional state of microglia changes at least twice. Already at middle age the cells are found in the reactive or immune alerted state, characterized by heightened Ca^2 +^ signaling but normal process motility whereas old mice harbor senescent microglia with decreased Ca^2 +^ signaling, and faster but disorganized directed movement of microglial processes. The 6–12 months long caloric restriction (70% of *ad libitum* food intake) counteracted these aging-induced changes shifting many but not all functional properties of microglia toward a younger phenotype. The improvement of Ca^2 +^ signaling was more pronounced in males. Importantly, even short-term (6-week-long) caloric restriction beginning at old age strongly improved microglial process motility and induced a significant albeit weaker improvement of microglial Ca^2 +^ signaling. Together, these data provide first sex-specific *in vivo* characterization of functional properties of microglia along the lifespan and identify caloric restriction as a potent, cost-effective, and clinically relevant tool for rejuvenation of microglia.

## Introduction

Aging is the main risk factor for neurodegenerative diseases (e.g., Alzheimer’s, Parkinson’s or Huntington’s disease), many of which have a clear gender-specific prevalence. Alzheimer’s disease (AD), for example, has a higher (1.6–3:1) prevalence in women compared to men, whereas Parkinson’s disease (PD) has a higher (3.5:1) prevalence in men compared to women (1). Moreover, aging is often accompanied by high blood pressure, obesity, physical inactivity, and unhealthy diet, all leading to accumulation of modified or displaced self molecules and activation of the immune system. Consistently, a persistent low grade inflammation, manifesting itself in increased levels of pro-inflammatory cytokines like tumor necrosis factor α (TNF-α), interleukin (IL) 6, IL-1β, and IL-18; upregulation of the expression levels of caspase-1, major histocompatibility complex, class II molecules (MHC-II), complement receptor 3 (CD11b) along with a concomitant decrease in the expression of anti-inflammatory factors such as IL-4 or brain-derived neurotrophic factor (BDNF) [reviewed in reference (2)], is a hallmark of the normally aging body and brain (3, 4). The transcriptome analyses of brains of cognitively normal subjects also revealed a profound change (mostly upregulation) in the expression of immune/inflammation-related genes with aging (5). Out of the 759 genes studied, major aging-dependent changes were seen in 40% of genes. Of note, much fewer genes (6% of immune-related probe sets) differed between AD patients and age-matched controls (5). Of these differentially expressed immune- and microglia-specific genes, many underwent just incremental changes (5), pointing to exaggerated response of immune system to aging as one of the key contributors to AD pathology (6, 7).

It is now generally accepted that with aging, microglia, the main immune cells of the brain, adopts so called “immune alerted” or “primed” phenotype, characterized by dystrophic morphology [i.e., increased soma volume, shorter and less complex processes and inhomogeneous tissue distribution (8–10)], reduced phagocytic capacity, elevated expression of inflammatory markers as well as an exaggerated and/or uncontrolled inflammatory response to immune stimuli (2, 4, 7, 11–13). Interestingly, recent *in vivo* data obtained in CX_3_CR1^*GFP*⁣/ +^ mice expressing eGFP in microglia (14), suggested that in the course of aging microglia change its phenotype at least twice. Already at middle age (9–11 months old mice) microglial phenotype switches from “homeostatic” to “immune alerted” or “reactive,” characterized by higher frequency and area under the curve of spontaneous Ca^2 +^ transients but normal motility of microglial processes, directed to the extracellular source of danger- or damage-associated molecular patterns (DAMPs). In old mice (18–21 months of age), microglia adopts “dysfunctional” or “senescent” phenotype, characterized by a high fraction of cells with spontaneous Ca^2 +^ signaling but diminished frequency and size of spontaneous as well as evoked Ca^2 +^ signals and fast but desynchronized DAMP-mediated movement of microglial processes. However, the mice used in the above study are partial knockouts for the fractalkine receptor CX_3_CR1 and therefore their “reactivity” might in part come from the diminished interaction between neuronal fractalkine and its microglial receptor CX_3_CR1. This interaction is known to represent a classical OFF signal, attenuating microglial reactivity (15, 16). Interestingly, dietary or caloric restriction (CR) is able to reduce inflammation in different species including humans (17) and is associated with reduced plasma levels of inflammatory markers (IL-6, C-reactive protein) as well as reduced up-regulation of genes for complement subunits, lysozyme, antigen presentation proteins, and other inflammation-related genes (4, 18). Still, how CR impacts *in vivo* functional properties of microglia remains unclear.

Furthermore, accumulating evidence suggests a complex interaction between the age and sex, especially in respect to brain’s immunocompetent cells like microglia. Indeed, sex steroids regulate the transcription of genes relevant for the development and maturation of the immune system (1) and MHC-II molecules, a hallmark of professional antigen-presenting cells, are higher expressed in male microglia both in man (19) and mice (20). Moreover, immune/inflammation-related genes respond in a gender-specific manner to aging and AD, with the most pronounced aging-related gender differences found in the hippocampus and the superior frontal gyrus (5, 21). Interestingly, while normal aging causes stronger enhancement of MHC-II expression in males, the opposite is true for AD patients, where the counts of MHC-II-expressing activated microglia were significantly higher in females (19). Despite the obvious importance of these data for the proper design of the preclinical and clinical studies, the *in vivo* functional properties of male and female microglia and their changes across the lifespan remain obscure.

By using *in vivo* two-photon imaging in young adult (2–4-month-old), middle-aged (9–11-month-old), and old (18–21-month-old) wild type (WT) mice, in the current study we asked how *in vivo* functional properties of microglia change in the course of aging and whether these age-dependent changes can be alleviated by caloric restriction.

## Materials and Methods

### Animals

This study included for both sexes three control groups of different age (2–4- (young), 9–11- (middle-age) and 18–21-month-old (old) wild type (C57BL/6) mice and three experimental (CR) groups age-matched with middle-age and old groups of control mice. In control groups 11 young adult, 10 middle-age and 14 old mice were used. The number of animals in CR groups is given below. The littermates were randomly assigned either to control or to the respective age-matched experimental group. Animals were kept under a 12 h’ light/dark cycle and were either fed *ad libitum* or followed one of the three different caloric restriction protocols (see below). All experimental procedures were in accordance with the Directive 2010/63/EU of the European Parliament and the Council of the European Union (see section “Ethics Statement”).

### Caloric Restriction

In preparation for the caloric restriction the individual daily *ad libitum* food intake of every mouse was measured over 14 days. The individual *ad libitum* food intake was used to calculate the exact amount of food the animal received during the CR. The food amount was gradually (over three weeks, 10% food reduction per week) reduced to 70% of the *ad libitum* food intake. The animals received the reduced food amount daily at the same time. CR mice were split into three groups based on the starting date and the duration of the CR diet: CR3 mice were under caloric restriction for at least 6 month starting from the age of 3 till 9–11 months (*n* = 7 mice); CR6 mice were under CR for at least 12 month from 6 till 18–21 months of age (*n* = 8 mice) and CR18 mice were under CR for 6 weeks starting at the age of 17–18 months (*n* = 10 mice). For all CR mice there was no return to *ad libitum* feeding before the end of experiment. In the control groups the animals had unlimited access to food. All mice were held in separate cages during the whole procedure. The body weight as well as the body status of every animal was monitored closely under the control of the animal doctors.

### *In vivo* Imaging of Microglia

Animal surgery was performed as described previously (22–24). Briefly, mice were anesthetized by isoflurane (induction: 2%, maintenance: 0.8–1%). Isoflurane concentration was adjusted such that the breathing rate was held between 90 and 140 BPM during the whole experiment. Body temperature was kept at 36–37°C. Skin removal was performed above the frontal cortex. The skull was cleaned, dried and a custom-made recording chamber with an opening in the middle was glued to the skull with cyanoacrylic glue (UHU, Baden-Baden, Germany). The skull located in the center of the opening was carefully thinned under a dissection microscope using a dental drill (Ultimate 500, NSK, Japan). After transfer into the imaging setup, the animal was positioned on a warming plate and the recording chamber was perfused with 37°C warm extracellular solution containing (in mM): 125 NaCl, 4.5 KCl, 26 NaHCO_3_, 1.25 NaH_2_PO_4_, 2 CaCl_2_, 1 MgCl_2_, 20 glucose, pH 7.4 when bubbled continuously with 95% O_2_ and 5% CO_2_. A craniotomy (∼1 mm^2^) was then performed above an area devoid of big blood vessels using a thin (30 G) syringe needle. Dura mater was left intact.

Imaging was performed using a two-photon laser-scanning microscope (Olympus Fluoview 300, Olympus, Tokyo, Japan) coupled to a mode-locked laser operating at 690 to 1040 nm wavelength (MaiTaiHP, SpectraPhysics, MountainView, CA, United States) and equipped with a 40× water-immersion objective (0.80 NA, Nikon, Tokyo, Japan). Oregon Green-BAPTA (OGB-1) and AlexaFluor 594 (AF594) were excited at a wavelength of 800 nm, the emitted light was split by a beam splitter (580 nm) and sent through the BP510/84 and BP630/92 filters, respectively.

### *In vivo* Labeling of Microglia

Microglial cells were visualized *in vivo* using tomato lectin conjugated to DyLight 594 (25). A glass micropipette filled with a tomato lectin (25 μm/ml) containing solution was inserted into the brain parenchyma via the craniotomy using a micromanipulator. The solution was injected into the cortical tissue by pressure application (40–55 kPa for 60 s). After ∼30 min wash-out time, this treatment resulted in labeling of microglia and endothelial cells in a tissue volume with the diameter of approximately 100–150 μm. The two cell types labeled were easily distinguished by morphology.

### *In vivo* Single-Cell Electroporation

Individual microglial cells were loaded with the small molecule Ca^2 +^ indicator Oregon Green BAPTA-1 (OGB-1) as described previously (22–24). In brief, a glass micropipette with a tip diameter of <1 μm was filled with 10 mM OGB-1 hexapotassium salt dissolved in a solution containing (in mM): 140 K-Gluconate, 14 KCl, 4 NaCl, and 10 HEPES, pH 7.3. DyLight 594-labeled microglial cells were approached with the micropipette using a manipulator (LN Junior, Luigs & Neumann). As soon as the micropipette touched the surface of the cell a negative current of 600 nA was applied for 10 ms (MVCS-02C iontophoresis system, NPI Electronic) and the pipette was immediately withdrawn.

### ATP Application

A total of 5 mM adenosine triphosphate (ATP) was dissolved in a standard solution of the following composition (in mM): 150 NaCl, 2.5 KCl and 10 HEPES, pH 7.4. The ATP-filled glass micropipette was positioned in the region of interest and a pressure of 15–30 kPa was applied for 50 ms to inject the ATP solution 30–40 μm away from the bodies of microglial cells of interest. To make pipette visualization easier, 200 μM of Alexa Fluor 594 was routinely added to the ATP-containing solution.

### Image Analyses

All image analyses were performed offline using Fiji^1^ and Igor Pro (Wavemetrics, Lake Oswego, OR, United States) software. To measure the Ca^2 +^-dependent changes in fluorescence, the region of interest (ROI) was drawn around the soma of the OGB-labeled microglial cell and the average fluorescence intensity within this area was determined. The average fluorescence intensity within a blood vessel area was used as background. The background-subtracted fluorescence intensity values were processed according to the formula ΔF/F = F/F_0_-1, where F_0_ is the basal level of fluorescence. Traces were filtered using a low-pass infinite impulse response filter with a cutoff frequency of 0.2 Hz. A change in fluorescence was defined as a Ca^2 +^ transient when its amplitude was higher than six times the standard deviation of the baseline noise. Each transient, whose fluorescence decayed to more than half of its maximum amplitude, was counted as a single event. Cells showing at least one Ca^2 +^ transient during a 15-min-long recording time were considered spontaneously active. The insert in Figure 2C shows how the amplitude, duration (T-half) and area under the curve (AUC) were measured.

To analyze the ATP-evoked process outgrowth, we used the procedures, developed previously (14). Briefly, maximum intensity projections (MIPs) of time-lapsed 3D stacks were acquired every 30 s over 15-min-long acquisition period at an x/y/z size of 141 μm × 141 μm × 20 μm with a step size of 2 μm. For analyses, an ellipse was fitted to the containment produced by microglial processes. The average diameter was determined by calculating the mean of the major and minor diameters at a given time point. The average diameter of the containment remained constant after the convergence of the microglial processes around the tip of the ATP-containing pipette. The mean of these values was considered as the final diameter of the containment. The containment formation velocity (μm/min) was calculated as the reduction of the average diameter over a given time. To evaluate the velocity of individual microglial process, the processes were manually tracked over time using the ImageJ plug-in “MTrackJ^2^”. For these analyses we only selected microglial processes whose tip could by unequivocally identified throughout at least seven consecutive time points. The average process velocity (μm/min) was calculated by measuring the average distance traveled by a tracked tip of the microglial process between the two consecutive time points.

### Analysis of the Single-Cell RNAseq Data

We analyzed single-cell RNAseq data of Bart De Strooper’s group (7), deposited in GEO under the GEO accession number: GSE127893. Only data sets belonging to cortical microglia from 3-month-old wild type C57BL/6 male and female mice were considered for analysis. Genes, differentially expressed in both sexes, were grouped according to gene ontology (GO) terms associated with inflammation and Ca^2 +^ signaling. We analyzed genes belonging to the following GO annotations: acute inflammatory response (GO:0002526), microglial cell activation (GO:0001774), positive regulation of inflammatory response (GO:0050729), release of sequestered calcium into cytosol (GO:0051209), calcium-mediated signaling using intracellular calcium source (GO:0035584), and positive regulation of cytosolic calcium ion concentration (GO:0007204). Another analyzed group contained all differentially expressed genes encoding potassium channels. Normalized gene expression values of significantly up- and down-regulated genes were plotted as median ± interquartile range (IQR). Comparison between males and females was performed using the Mann-Whitney test.

### Statistics

For each experiment, the choice of the sample size was based on biometrical sample size estimation. GraphPad Prism 6 (GraphPad Softwar Inc., La Jolly, CA, United States) or MATLAB (MathWorks, Inc., Natick, MA, United States) were used to perform all statistical analyses. The one-sample Kolmogorov-Smirnov test was used to check for normality of the data distribution. The Tukey method in MATLAB (function “Quartiles”) was used to identify outliers. Unless otherwise indicated, all data are given as median ± interquartile range (IQR). Lines of boxes and whiskers represent 25th and the 75th (boxes) and 10th and 90th (whiskers) percentile.

To compare the different age groups the Kruskal-Wallis test followed by Dunn’s *post hoc* test for multiple comparisons was used, for the comparison between control and CR mice the Mann-Whitney test was performed. Statistical interactions between the age and sex were determined by the two-way ANOVA followed by the Bonferroni’s *post hoc* test.

## Results

### Age-Dependent Changes of *in vivo* Microglial Ca^2 +^ Signaling in WT Mice

**FIGURE 1:**
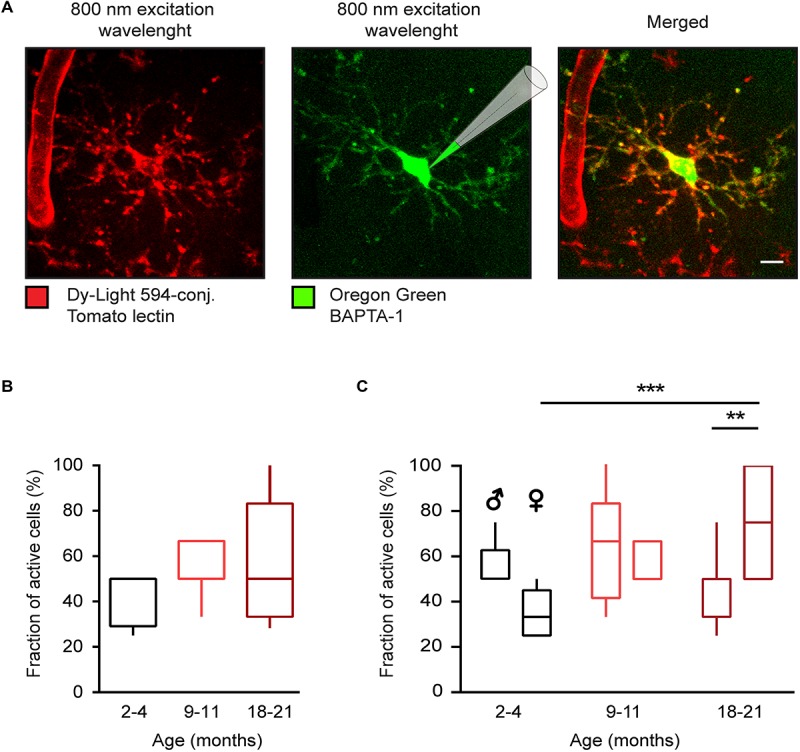
Age-dependent changes of *in vivo* microglial Ca^2 +^ signaling in WT mice. **(A)** Maximum intensity projection (MIP) images (110–126 μm below the cortical surface, here and below the step size is 1 μm) of a representative microglial cell in a WT mouse labeled with the DyLight 594-conjugated Tomato lectin (left) and loaded with the small molecule Ca^2 +^ indicator Oregon Green BAPTA-1 (OGB-1) by means of single-cell electroporation (middle). Merged image is shown on the right. **(B)** Box-and-whisker plot illustrating the fractions (per mouse) of spontaneously active microglia in 2–4- (*n* = 11 mice, 39 cells), 9–11- (*n* = 10 mice, 34 cells), and 18–21- (*n* = 14 mice, 46 cells) month-old mice. **(C)** Box-and-whisker plot illustrating the effect of the age and sex on the fraction (per mouse) of spontaneously active microglia [*n* = 5 males (16 cells), 6 females (23 cells) for 2–4 months old mice; 5 males (18 cells), 5 females (16 cells) for 9–11 months old mice and 7 males (23 cells), 7 females (23 cells) for 18–21 months old mice]. Statistical differences were determined using two-way ANOVA followed by Bonferroni’s *post hoc* test (**p < 0.01 and ***p < 0.001). Scale bar, 5 μm.

To study age- and sex-related differences in the spontaneous Ca^2 +^ signaling of microglia, cells in the layer 2–3 of the frontal cortex (Figure 1A) were visualized by means of *in vivo* staining with DyLight 594-conjugated Tomato lectin (25, 26) and loaded with the small molecule Ca^2 +^ indicator OGB-1 via single cell electroporation (22, 24). We analyzed three different age groups of WT mice: 2–4, 9–11, and 18–21 months old, designated as “young adult”, “middle-age” and “old” mice, respectively. Microglial cells were considered spontaneously active, if they showed at least one Ca^2 +^ transient (recorded as described in the section “Materials and Methods”) during a 15-min-long recording period. In the young adult group the fraction (per mouse) of spontaneously active microglial cells was higher than found previously in age-matched CX_3_CR1^*GFP*⁣/ +^ mice (14, 22). To test whether this discrepancy is caused by sex differences, we separated the data obtained in male and female mice. Indeed, there was a significant difference in the fraction of active cells between young adult male [50 ± 12.5% (median ± IQR)] and female (33.3 ± 20%) mice (*p* = 0.04, Mann-Whitney test).

To test how our *in vivo* data relate to the transcriptome differences between male and female microglia, we made use of the recently published data set available under the accession number GEO: GSE127893 and containing, amongst others, single-cell RNAseq data from 3-month-old male and female WT mice (7). Consistent with other recent data (20), inflammation-related genes were significantly upregulated in young adult male compared to female mice (Supplementary Figure 1A), along with significantly higher expression levels of genes encoding Kir, K_2__*P*_, and Kv potassium channels (Supplementary Figure 1B). The Ca^2 +^ related pathways in general, as well as biological processes summarized under GO annotations “release of sequestered calcium into cytosol,” “calcium-mediated signaling using intracellular calcium source,” or “positive regulation of cytosolic calcium ion concentration” (see Supplementary Table S1 for list of included genes) were all upregulated in young adult male compared to female mice but the difference observed did not reach the level of statistical significance (Supplementary Figures 1C–F).

Next, we tested how the fraction of active cells changed as the animals aged (Figures 1B,C). In the lumped data, there was only a slight trend toward an increase in the fraction of active cells with age (Figure 1B), in contrast to data obtained in CX_3_CR1^*GFP*⁣/ +^ mice (14, 22). This discrepancy is likely explained by the fact that although in females the fraction (per mouse) of active cells gradually increased with age amounting to 75.00 ± 50% in old female mice and thus being significantly different from fraction measured in young adult females (*p* < 0.001, Two-way ANOVA followed by Bonferroni’s *post hoc* test), this was not the case for male mice. Here, the fraction of active cells did not change significantly over time, showing, if at all, a trend toward a decrease with age (50 ± 12.5, 66.67 ± 41.7, and 33.3 ± 50% for young adult, middle-aged and old mice, respectively). Consistently, there was a significant difference in the fraction of active cells between old males and females (*p* = 0.002, Two-way ANOVA followed by Bonferroni’s *post hoc* test).

Thus, when looking on young adults, there is a significant difference in the fraction of spontaneously active cells between the male and female mice. This difference is consistent with increased inflammatory milieu found in the transcriptome analysis of male microglia and likely reflects an increased alertness of these cells and the higher sensitivity to minute damages in their microenvironment (see section “Discussion”). Aging, however, impacts significantly more on female microglia, turning spontaneously active the vast majority of these cells.

### Age-Dependent Changes in the Time Course of Spontaneous Ca^2 +^ Transients

**FIGURE 2:**
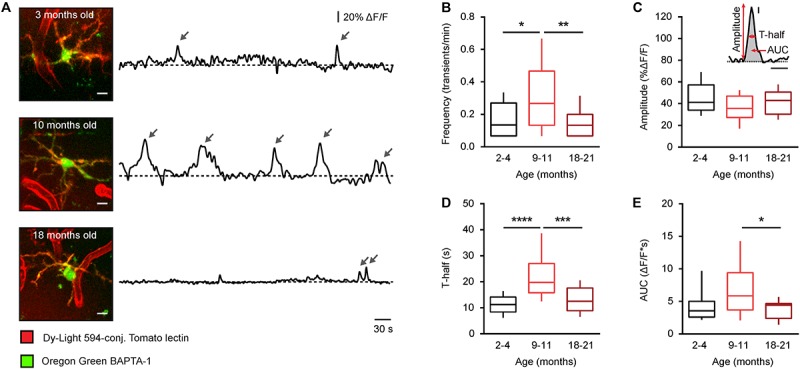
Age-dependent changes of the time course of somatic Ca^2 +^ signals in microglia from WT mice. **(A)** MIP images of representative microglial cells from 2– 4-, 9– 11-, and 18–21-month-old mice (left; 82–103 μm, 73–97 μm, and 65–87 μm below the cortical surface, respectively) as well as spontaneous Ca^2 +^ transients (arrows) recorded from these cells *in vivo* during a 15-min-long recording period (right). Scale bar, 5 μm. **(B–E)** Box-and-whisker plots illustrating the median (per cell) frequency **(B)**, amplitude **(C)**, T-half **(D),** and AUC **(E)** of spontaneous Ca^2 +^ transients in microglia from 2–4- (*n* = 19 cells), 9–11- (*n* = 19 cells), and 18–21- (*n* = 28 cells) month-old mice. Inset in **(C)** shows an example of a Ca^2 +^ transient and schematically illustrates the parameters analyzed for each Ca^2 +^ transient. Inset scale bars, 5% ΔF/F and 10 s. Statistical differences were determined using Kruskal-Wallis test followed by Dunn’s *post hoc* test for multiple comparisons (*p < 0.05, **p < 0.01, ***p < 0.001, and ****p < 10^−4^).

To further analyze the aging-dependent changes, different properties of the spontaneous microglial Ca^2 +^ transients, exemplified for each age in Figure 2A, were evaluated in detail (Figure 2): the frequency (transients/min), the amplitude (% of ΔF/F), the duration (T-half), and the area under the curve (AUC, ΔF/F^∗^s). The frequency plot had a bell-shaped appearance with a significantly higher frequency observed in middle-aged compared to young adult and old mice (*p* = 0.04 and *p* < 0.01, respectively, Kruskal-Wallis test followed by Dunn’s *post hoc* test for multiple comparisons, Figure 2B), whereas the amplitude showed no difference between the three different age groups (*p* = 0.16, Kruskal-Wallis test, Figure 2C). The duration (T-half) of the Ca^2 +^ transients was significantly higher in middle-aged compared to young adult and old mice (*p* < 0.001 for both comparisons, Kruskal-Wallis test followed by Dunn’s *post hoc* test, Figure 2D). The AUC showed a similar bell-shaped trend like the frequency and the T-half but the observed difference reached the level of statistical significance only for comparison between the middle-aged and the old mice (*p* = 0.04, Kruskal-Wallis test followed by Dunn’s *post hoc* test, Figure 2E).

Thus, consistent with our data obtained in CX_3_CR1^*GFP*⁣/ +^ mice (14), the microglia in middle-aged mice have a significantly heightened Ca^2 +^ signaling compared to microglia in both young adult and old mice.

### Sex-Specificity of the Age-Dependent Changes in the Time Course of Spontaneous Ca^2 +^ Transients

**FIGURE 3:**
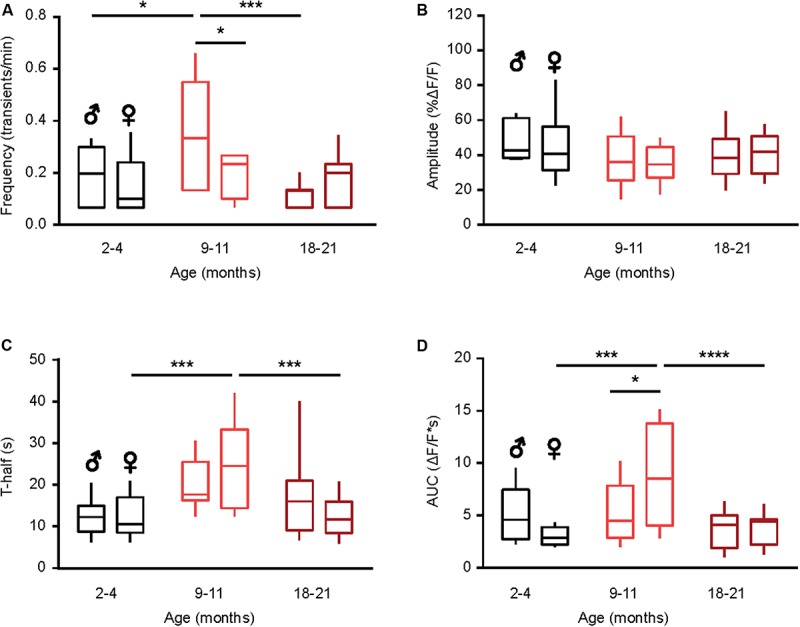
Sex-specificity of the time course of spontaneous Ca^2 +^ transients and its dependence on animal‘s age. **(A–D)** Box-and-whisker plots illustrating the effect of age and sex on the median (per cell) frequency **(A)**, amplitude **(B)**, T-half **(C)** and AUC **(D)** of the spontaneous Ca^2 +^ transients in microglia [*n* = 9 cells (males), 10 cells (females) for 2–4 months old mice; 10 cells (males), 9 cells (females) for 9–11 months old mice and 10 cells (males), 18 cells (females) for 18–21 months old mice]. Statistical differences were determined using two-way ANOVA followed by Bonferroni’s *post hoc* test (*p < 0.05, ***p < 0.001, and ****p < 10^–^^4^).

Next, we studied the sex-specificity of the different properties of spontaneous Ca^2 +^ transients during aging. Males showed a bell-shaped distribution for the frequency of spontaneous Ca^2 +^ transients (Figure 3A) with a significantly higher frequency in the middle-aged compared to young adult and old mice (*p* = 0.01, *p* < 0.001 for comparisons between young and middle-aged and middle-aged and old, respectively, Two-way ANOVA followed by Bonferroni’s *post hoc* test). In contrast, the frequency did not change with age in microglia from female mice (*p* > 0.99 for all comparisons, Two-way ANOVA followed by Bonferroni’s *post hoc* test). Consistently, a significantly higher frequency was observed in male compared to female mice of the middle-age group (*p* = 0.03, Two-way ANOVA followed by Bonferroni’s *post hoc* test; Figure 3A). We did not observe any sex- or age-dependent differences for the amplitude of spontaneous Ca^2 +^ transients (*p* > 0.1 for all comparisons, Two-way ANOVA, Figure 3B). For the duration (T-half) the graph (Figure 3C) had a bell shape for both sexes but only for females the durations of Ca^2 +^ transients in the middle-aged mice were significantly higher compared to young adult and old animals (*p* < 0.001 for both comparisons, Two-way ANOVA followed by Bonferroni’s *post hoc* test). Similar data were obtained for the AUCs of Ca^2 +^ transients in female mice (*p* < 0.001 for both comparisons, Two-way ANOVA followed by Bonferroni’s *post hoc* test; Figure 3D), whereas for male mice we did not observe any sex- or age-dependent differences. Concordantly, the AUCs measured in middle-aged females were significantly higher than the ones measured in the age-matched males (*p* = 0.03, Two-way ANOVA followed by Bonferroni’s *post hoc* test, Figure 3D).

Taken together, these data further emphasize the “immune alerted” or “reactive” phenotype of microglia in middle-aged mice of both sexes but also reveal several sex-specific differences in the age-dependent changes of microglial Ca^2 +^ signaling.

### Effect of Caloric Restriction on the Properties of Spontaneous Ca^2 +^ Transients

**FIGURE 4:**
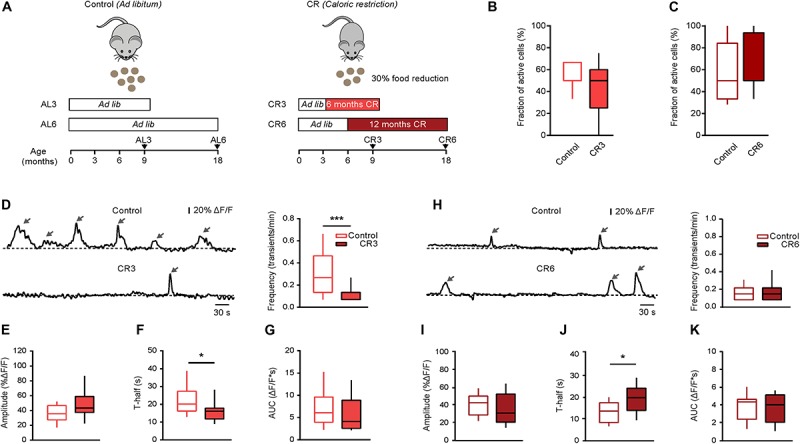
Effect of caloric restriction on spontaneous Ca^2 +^ signals in middle-aged and old mice. **(A)** Schematic description of caloric restriction protocols used in these experiments. In the control group (left), the animals had unlimited access to food (*ad libitum*). In the CR group (right), mice were daily fed with the 70% of their respective initial *ad libitum* intake. CR mice were split into two groups based on the starting date and the duration of the CR diet: CR3 mice were under CR from 3 till 9 months of age; CR6 mice were under CR from 6 till 18 months of age. For all CR mice there was no return to *ad libitum* feeding before the end of experiment. **(B)** Box-and-whisker plot illustrating the fractions (per mouse) of spontaneously active microglia in 9–11 months old control (*n* = 10 mice, 34 cells) vs. CR3 (*n* = 7 mice, 26 cells) mice. **(C)** Box-and-whisker plot illustrating the fractions (per mouse) of spontaneously active microglia in 18–21 months old control (*n* = 14 mice, 46 cells) vs. CR6 (*n* = 8 mice, 26 cells) mice. **(D,H)** Left: representative spontaneous microglial Ca^2 +^ transients (arrows) measured in control (top) and CR (bottom) mice. Right: box-and-whisker plots illustrating the median (per cell) frequency of spontaneous Ca^2 +^ transients in microglia from the respective groups. **(E–K)** Box-and-whisker plots illustrating the median (per cell) amplitude **(E,I)**, T-half **(F,J)** and AUC **(G,K)** of the spontaneous Ca^2 +^ transients in control vs. CR mice (*n* = 19 cells (control), 12 cells (CR3) for 9–11 months old mice; *n* = 28 cells (control), 16 cells (CR6) for 18–21 months old mice). Statistical differences were determined using Mann-Whitney test (*p < 0.05, ***p < 0.001).

In the next series of experiments, we tested whether reducing the food intake can counteract the aging-related changes in microglial Ca^2 +^ signaling. First, we used long lasting (CR3 and CR6) caloric restriction protocols described in detail in the section “Materials and Methods.” Briefly, the food intake of every animal was monitored over two weeks to determine the average daily *ad libitum* food intake. In the first CR group (CR3), the daily food intake was slowly reduced to 70% of the average *ad libitum* food intake at the age of 3 months and was kept at this level for at least 6 months. All experiments for the CR3 group were performed in mice between 9 and 11 months of age (i.e., in middle-aged mice). After 6 months of CR, the body weight of CR3 mice (88.4 ± 4.6% of the initial weight at 3 months of age) differed significantly from that of age-matched *ad libitum* fed control mice, which increased their body weight (138.2 ± 20.2%) during the same time period (*p* < 0.001, Mann-Whitney test, *n* = 7 CR3 and 10 control mice). In the second CR group (CR6), the daily food intake was slowly reduced to 70% of the average *ad libitum* food intake at the age of 6 months and was kept at this level for at least 12 months. The experiments for the CR6 group were performed at the age of 18 to 21 months (i.e., in old mice, Figure 4A). After 12 months of CR, the body weight of CR6 mice (81.3 ± 8.8% of the initial weight at 6 months of age) also differed significantly from that of age-matched *ad libitum* fed control mice, which increased their body weight (116.5 ± 30.7%) during the same time period (*p* < 0.001, Mann-Whitney test, *n* = 8 CR6 and 14 control mice). Compared to *ad libitum* fed age-matched mice, neither CR protocol caused a significant change in the fraction (per mouse) of spontaneously active microglial cells (*p* = 0.25 for CR3 and *p* = 0.67 for CR6 protocol, Mann-Whitney test; Figures 4B,C). However, the median frequency of spontaneous Ca^2 +^ transients in CR3 mice decreased significantly, compared to that measured in *ad libitum* fed control mice (*p* < 0.001, Mann-Whitney test; Figure 4D) becoming similar to the frequency of spontaneous Ca^2 +^ transients measured in young adult mice (*p* = 0.17, Mann-Whitney test). Similar effect of CR3 was observed for the duration (T-half) of the spontaneous Ca^2 +^ transients (*p* = 0.03 when comparing CR3 with *ad libitum* fed middle-age mice; Figure 4F). For T-half, however, the difference between CR3 with *ad libitum* fed young adult mice still remained significant (*p* = 0.03, Mann-Whitney test). No significant effects of CR3 were observed for amplitudes (*p* = 0.1, Mann-Whitney test; Figure 4E) and the AUCs (*p* = 0.19, Mann-Whitney test; Figure 4G) of the spontaneous Ca^2 +^ transients.

In contrast to the CR3 mice, no significant change was observed in the frequency of the spontaneous Ca^2 +^ transients in CR6 compared to age-matched *ad libitum* fed mice (*p* = 0.9, Mann-Whitney test; Figure 4H). Similarly, CR6 influenced neither the amplitudes (*p* = 0.18, Mann-Whitney test; Figure 4I) nor the AUCs (*p* = 0.96, Mann Whitney test; Figure 4K) of the spontaneous Ca^2 +^ transients. Only the duration (T-half) of the spontaneous Ca^2 +^ transients showed a significant increase in comparison to age-matched *ad libitum* fed mice (*p* = 0.01, Mann Whitney test, Figure 4J), becoming increasingly similar to the T-half measured in middle-aged mice (*p* = 0.25 when comparing CR6 with *ad libitum* fed middle-aged mice, Mann-Whitney test).

In summary, reduction of the daily food intake by caloric restriction changes *in vivo* functional properties of microglial Ca^2 +^ signals, making them more similar to the properties observed in the younger age group.

### Sex-Specific Sensitivity to Caloric Restriction

**FIGURE 5:**
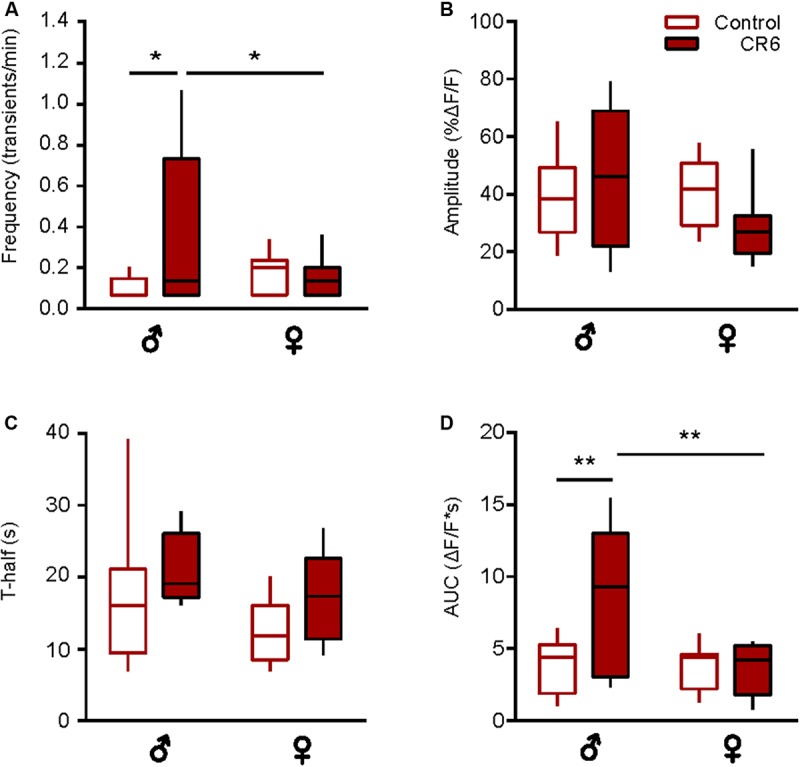
Sex-specific effect of caloric restriction on the time course of spontaneous Ca^2 +^ transients. Box-and-whisker plots illustrating the sex-specific effects of CR6 on the median (per cell) frequency **(A)**, amplitude **(B)**, T-half **(C),** and AUC **(D)** of spontaneous Ca^2 +^ transients [*n* = 10 cells (7 males), 18 cells (7 females) for control and *n* = 5 cells (3 males), 11 cells (5 females) for CR6 groups]. Statistical differences were determined using two-way ANOVA followed by the Bonferroni’s *post hoc* test (*p ≤ 0.05, **p < 0.01).

Next, we analyzed the sex-specific effects of CR. Compared to middle-aged *ad libitum* fed male mice (Supplementary Figure 2), CR3 induced a significant reduction in the frequency of spontaneous Ca^2 +^ transients (*p* < 0.01, Mann-Whitney test) and an almost significant reduction in T-half (*p* = 0.05, Mann-Whitney test), thus counteracting all aging-related changes observed in this age group (compare to Figure 3). Because of the unexpected death of several female mice, it was unfortunately not possible to analyze CR3 females separately. Compared to old *ad libitum* fed mice, there was no sex-specific influence of CR6 on the fraction of active microglial cells (*p* > 0.5 for all comparisons, Chi-Square test, *n* = 23 cells (7 females), 23 cells (7 males) for control mice and *n* = 17 cells (5 females), 9 cells (3 males) for CR6 groups). In male mice, CR6 caused a significant increase in the frequency (*p* = 0.02, Two-way ANOVA and Bonferroni’s *post hoc* test; Figure 5A) and AUC (*p* < 0.01, Two-way ANOVA and Bonferroni’s *post hoc* test; Figure 5D) of spontaneous Ca^2 +^ transients. The other two parameters analyzed (Figures 5B,C) were not influenced by caloric restriction (*p* = 0.9 and 0.6 for the amplitude and T-half, respectively, Two-way ANOVA and Bonferroni’s *post hoc* test). There was, however, a small but consistent trend toward an increase in T-half in mice of both sexes, resulting in a significant change in the combined dataset (Figure 4J). Yet, in female mice we did not observe any significant CR-induced changes (Figure 5). Such CR-resistance of female mice led to significant differences between CR-treated males and females with regard to the frequency and the AUC of spontaneous Ca^2 +^ transients (*p* = 0.05 and *p* < 0.01, respectively, Two-way ANOVA and Bonferroni’s *post hoc* test; Figures 5A,D).

Together, these data support the “rejuvenating” effect of CR on the microglial Ca^2 +^ signaling. At the same time, they also reveal the higher CR-sensitivity of male microglia.

### Effect of Caloric Restriction on Aging-Mediated Changes in Process Chemotaxis

**FIGURE 6:**
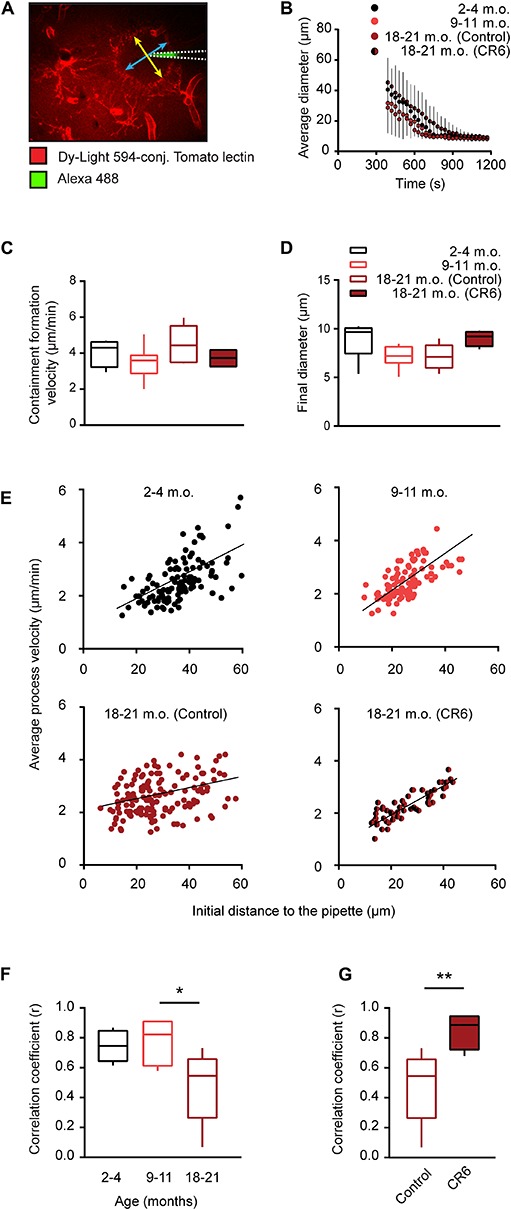
Effect of caloric restriction on ATP-induced process chemotaxis of microglia. **(A)** MIP image of a Z stack (80–100 μm below the cortical surface, 2 μm step size) exemplifying the containment formation by microglial processes labeled with DyLight 594-conjugated Tomato lectin in a 3-month-old mouse. The stack was taken 10 min after pressure application (50 ms, 30 kPa) of 5 mM ATP. Yellow and blue arrows indicate the major and minor diameters of the containment, respectively. White dashed lines emphasize the location of the ATP-containing pipette. **(B)** Graph illustrating changes in the average diameter of the containment over time and the effect of CR. The moment of the ATP application was taken as time point 0. **(C,D)** Box-and-whisker plots illustrating the median (per mouse) containment formation velocity **(C)** and the final diameter of the containment **(D)** in 2–4- (*n* = 7 mice), 9–11- (*n* = 8 mice), 18–21- (*n* = 8 mice) month-old control mice, and in 18–21- (*n* = 4 mice) month-old CR6 mice. **(E)** Scatter plots illustrating the relationship between the initial distance of a microglial process to the tip of the ATP-containing pipette (*X*-axis) and the mean velocity of the process (*Y*-axis) in 2–4- (*n* = 6 mice, 118 processes), 9–11- (*n* = 6 mice, 119 processes), 18–21- (*n* = 8 mice, 146 processes) month-old control mice as well as in 18–21- (*n* = 4 mice, 58 processes) month-old CR6 mice. **(F,G)** Box-and-whisker plot illustrating median (per mouse) Spearman’s correlation coefficients between the initial distance of microglial processes to the tip of the ATP-containing pipette and the mean process velocity in the three age groups **(F)** as well as the 18–21 months old control and age-matched CR6 groups **(G)**. Statistical differences between age groups were determined using Kruskal-Wallis test followed by Dunn’s *post hoc* test for multiple comparisons. For comparing control and CR6 mice Mann-Whitney test was used (*p < 0.05, **p < 0.01).

Tissue injury in the local microenvironment of microglia causes an extension of microglial processes toward the site of injury aiming at insulation of the damaged site from the intact brain parenchyma (27). This tissue-protective response of microglia can also be triggered by a local application of ATP from a micropipette (27), as tissue damage is always accompanied by ATP release from injured cells. Therefore, ATP is considered a classical DAMP (28). Under such conditions microglial processes, located in the vicinity of the micropipette (containing in our case 5 mM ATP), rapidly extend toward the tip of the pipette forming a spherical containment (Figure 6A). To characterize the ATP-induced microglial process extension in the three age groups under study, we first monitored over time the average diameter of the containment formed by the processes around the ATP-containing pipette. The mean diameter of the containment decreased faster as the animals were getting older (Figure 6B). The fastest decrease was observed in old mice. Interestingly, this effect was averted by the caloric restriction (CR6) so that the time courses measured in CR6 and young adult mice looked similar (Figure 6B). Consistently, the containment formation velocity increased with age (Figure 6C) but, in contrast to data obtained for CX_3_CR1^*GFP*⁣/ +^ mice (14), in WT mice studied here this effect did not reach the level of statistical significance (*p* = 0.17, Kruskal-Wallis test). Similarly, we did not observe any significant difference for the final diameter of the spherical containment surrounding the tip of the pipette between the three control age-groups as well as CR6 mice (*p* = 0.1 Kruskal-Wallis test; Figure 6D), indicating that in all groups studied roughly equal number of microglial processes contribute to the containment formation.

Further, we analyzed the velocity of individual microglial processes moving toward the tip of the pipette (see section “Materials and Methods” for details) and plotted it versus the initial distance between the process tip and the pipette (Figure 6E). Similar to data obtained for CX_3_CR1^*GFP*⁣/ +^ mice (14), there was a strong positive correlation between the two parameters in young adult and middle-aged mice, reflected by a median (per mouse) Spearman’s rank correlation coefficient (r) of 0.75 for young adult and 0.82 for middle-aged mice (Figure 6E). A significant and sex-independent decrease in the correlation was observed in old (*r* = 0.55) compared to middle-aged mice (*p* = 0.04, Kruskal-Wallis test followed by Dunn’s *post hoc* test; Figure 6F and Supplementary Figure 3). Importantly, this decrease was abrogated by the caloric restriction (CR6; Figure 6E), causing a significant increase in the correlation coefficient (*p* < 0.01 for comparison between old and CR6 mice, Mann Whitney test; Figure 6G).

Thus, in old mice of both sexes the microglial processes move faster but more disorganized toward the nearby DAMP source, compared to young adult and middle-aged mice. This age-induced dyscoordination of the process movement is counteracted by the long lasting caloric restriction.

### Positive Effects of Short-Term Caloric Restriction in Old Mice

**FIGURE 7:**
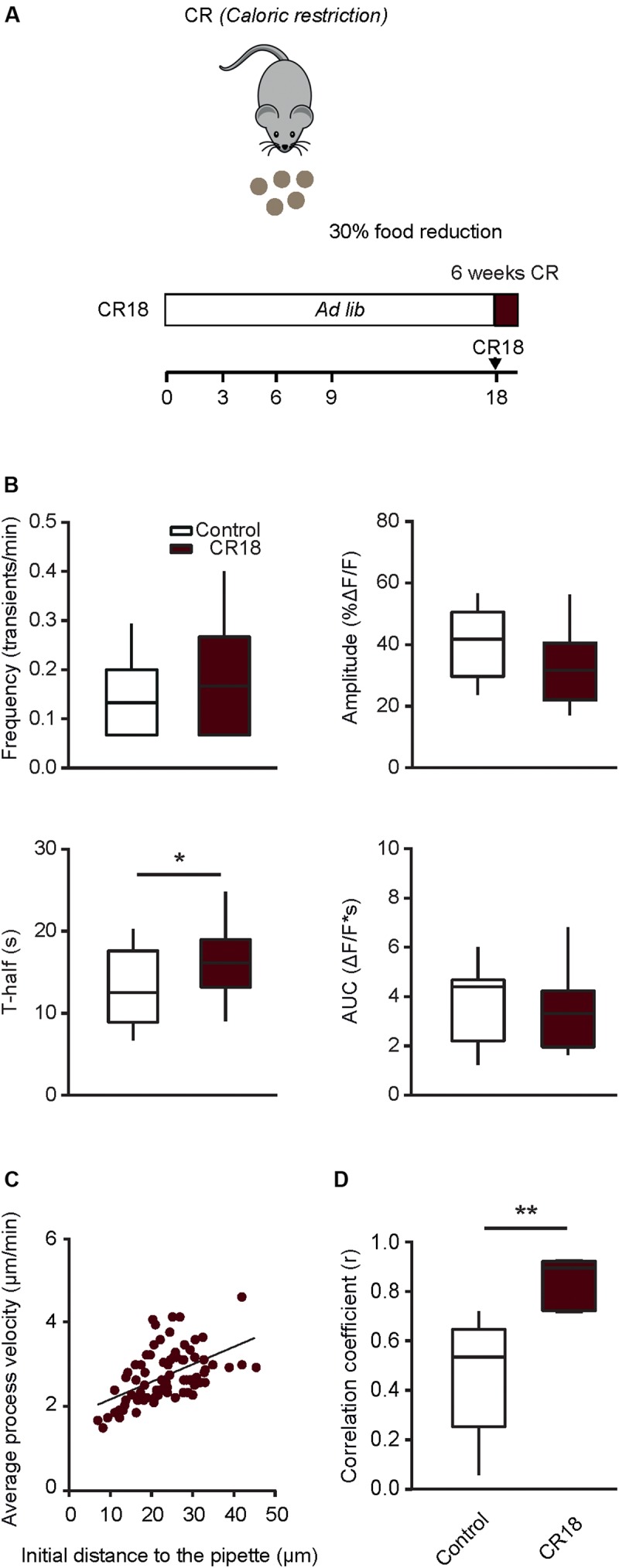
Short-term caloric restriction in old mice induces a partial shift of functional properties of microglia toward a younger phenotype. **(A)** Schematic description of the 6-week-long caloric restriction protocol (CR18) used in old mice. **(B)** Box-and-whisker plots illustrating the median (per cell) frequency (top left), amplitude (top right), T-half (bottom left) and AUC (bottom right) of spontaneous Ca^2 +^ transients recorded in microglia from 18 to 21 months old control (*n* = 28 cells, 14 mice) and CR18 mice (*n* = 19 cells, 10 mice). **(C)** Scatter plot illustrating the relationship between the initial distance of a microglial process to the tip of the ATP-containing pipette (*X*-axis) and the mean velocity of the process (*Y*-axis) in CR18 mice (*n* = 80 processes, 5 mice). **(D)** Box-and-whisker plot illustrating median (per mouse) Spearman’s correlation coefficients between the initial distance of microglial processes to the tip of the ATP-containing pipette and the mean process velocity in 18–21 months old control (*n* = 8 mice) vs. CR18 (*n* = 5 mice) mice. Statistical differences were determined using Mann-Whitney test (*p < 0.05, **p < 0.01).

In order to test whether a long lasting CR is crucial for inducing the positive effects described above, in the next step we applied a short-term CR protocol (CR18). In the CR18 group, the daily food intake was slowly reduced to 70% of the average *ad libitum* food intake at the age of 17 to 18 month and the experiments were performed in 19 month-old mice, after 6–7 weeks of 70% CR (Figure 7A). This short-term CR led to a significant decrease in body weight (79.0 ± 10.2% of the initial weight before the start of CR), compared to that of age-matched *ad libitum* fed control mice, which, however also lost some weight during the observation time (93.9 ± 5.7%; *p* = 0.01, Mann-Whitney test, *n* = 10 CR18 and 6 *ad libitum* fed mice). The median (per mouse) fraction of spontaneously active cells showed no significant difference between CR18 and the age matched control mice (*p* = 0.1, Mann-Whitney test). Analyses of the frequency and the time course of spontaneous Ca^2 +^ transients (Figure 7B) revealed no significant difference between CR18 and age-matched control animals for all parameters tested besides T-half (*p* = 0.048 for T-half, *p* > 0.05 for all other comparisons, Mann-Whitney test). When analyzing the above parameters we also did not observe any significant sex-specific effects of CR18 (*p* > 0.09 for all comparisons, Two-way ANOVA followed by the Bonferroni’s *post hoc* test).

Interestingly, however, the ATP-induced movement of individual microglial processes (Figure 7C) was much more coordinated in CR18 compared to old control mice, yielding the median (per mouse) Spearman’s rank correlation coefficient (r) of 0.9, which differed significantly from that found in age-matched control mice (*p* = 0.003, Mann-Whitney test, Figure 7D).

Thus, short term caloric restriction conducted in old mice, i.e., after the ageing-induced microglial dysfunction has already occurred, recapitulated the major and most robust effects of long-term CR (compare Figures 4J, 6E–G, 7) but did not induce more subtle sex-specific changes shown in Figure 5.

## Discussion

Understanding the functional heterogeneity of microglial cells is one of the key aims of modern neurobiology. The importance of this aim is underscored by the multitude of functions these cells assume under homeostatic as well as pathological conditions. Accumulating evidence suggests that both organism’s sex and age are critical determinants of the functional state of microglia. So far, however, the supporting evidence came either from “omics” studies (7, 9, 20, 21, 29) or from *in vitro/in vivo* imaging of microglial protein makeup, morphology or surveillance (8, 10, 30–33). Our study, addressing *in vivo* microglial Ca^2 +^ signaling as well as DAMP-evoked directed process motility, adds important functional aspects to this knowledge.

Although we did not evoke the microglial Ca^2 +^ signals under study experimentally and therefore have to name them “spontaneous,” we and others have shown that such Ca^2 +^ signals are only seen in the presence of local [e.g., damage of cell/tissue or accumulation of amyloid β in microglial vicinity (22, 23, 34, 35)] or global [e.g., systemic inflammation (34, 36)] threats and thus likely reflect the activation of microglia as well as their reaction to pathogen- or danger-associated signals.

Our *in vivo* analyses revealed that (i) the fraction of spontaneously active and therefore likely alerted microglial cells is significantly higher in young adult male mice; (ii) normal aging, however, much stronger increases the fraction of active cells in females; (iii) when considering both the fraction of active cells and the frequency/time course of Ca^2 +^ transients, the most alerted (reactive) microglia is found in middle-aged and not, as assumed by the literature, old mice; (iv) Ca^2 +^ signals recorded in middle-aged mice carry a peculiar sex-specific signature with males having significantly higher frequency and females – significantly larger size of Ca^2 +^ transients; (v) aging causes a dyscoordination of directed movement of microglial processes, common for both sexes. Moreover, prolonged (6–12 months long) caloric restriction causes (vi) rejuvenation of microglial Ca^2 +^ signals, shifting their functional properties toward those observed in younger age groups and (vii) re-synchronizes directed DAMP-induced movement of individual microglial processes. Interestingly, (viii) even a short-term (6-weeks-long) caloric restriction conducted in old age is able to improve the duration of Ca^2 +^ transients and microglial process coordination significantly, thus paving a way for development of new, cost-effective and widely accessible anti-inflammatory therapies in elderly.

### Higher Fraction of Active Cells in Young Adult Males

Our experiments conducted in DyLight 594-conjugated Tomato lectin-labeled microglia from young adult mice provided a somewhat higher fraction of spontaneously active cells compared to data obtained previously by us and others (14, 22, 23, 34, 35). Besides the differences among the mouse strains used (i.e., WT vs. CX_3_CR1^*GFP*⁣/ +^ vs. Iba-1^*GFP*⁣/ +^, etc.), one logical explanation would be that the extracellular binding of lectin molecules might aggravate the diffusion of OFF molecules to their microglial receptors, as the reduction of the surrounding neuronal activity is known to increase spontaneous Ca^2 +^ signaling in microglia (23). However, similar fractions of active cells were observed in Tomato lectin-labeled WT and CX_3_CR1^*GFP*⁣/ +^ microglia from 9 to 11 months and 18 to 21 months old mice [this study and reference (14)] and we failed to increase the frequency of spontaneous Ca^2 +^ transients in young adult CX_3_CR1^*GFP*⁣/ +^ microglia upon injection of DyLight 594-conjugated Tomato lectin (data not shown). Therefore, the most likely explanation remains the different fraction of male and female mice in the combined dataset.

Interestingly, recent “omics” data showed that in young adult male mice microglia upregulate genes belonging to Gene Ontology classes associated with inflammatory processes, including regulation of cell migration and cytokine production [reference (37) and our analysis of data obtained by Bart De Strooper’s group (7)]. Moreover, our analysis showed significant upregulation of genes encoding K^+^ channels in male mice, including genes belonging to both Kir/K_2__*P*_ and Kv families. Consistently, in cortical slices male microglia had higher baseline outward and inward currents and responded with larger inward K^+^ currents to applications of ATP (20). Because an increase in the K^+^ current density is a known sign of microglial activation (38), together these data firmly document higher reactivity of young male microglia. The higher spontaneous Ca^2 +^ signaling observed in these cells *in vivo* is likely caused by the increased driving force for Ca^2 +^ ions flowing through the plasma membrane (due to K^+^ channel-induced cell hyperpolarization) as well as, according to our GO analysis, enhanced Ca^2 +^ release from the intracellular Ca^2 +^ stores.

### Faster Aging of Microglial Population in Female Mice

While starting at the higher level in young adult mice, the fraction of active cells in males changed little over the lifespan, much in contrast to female mice, in which this fraction roughly doubled within the same time window. These functional data are consistent with recent single-cell analyses of microglial transcriptome (7) and point to “faster aging” of female microglia. On the mRNA level this process is accompanied by an earlier upregulation of gene sets involved in antigen presentation, innate immune response or other inflammatory processes as well as interferon response type I pathways (7). The upregulated genes, however, also included genes likely involved in tissue regeneration (e.g., Spp1, Gpnmb, and Dkk2), so that the overall functional outcome of the faster aging of microglial population in female mice still remains to be determined. It has to be also noted that on the single cell level we did not observe any difference between the frequency as well as the time course of Ca^2 +^ signals in aged male and female mice.

### Middle Age as the First Turning Point in Microglial Homeostasis

Whereas genomic, proteomic as well as morphological analyses clearly document phenotypic differences between the old and the young adult microglia [reviewed in refs. (2, 4, 12, 39)], the middle-aged microglia is much less characterized. At the same time, many neuroinflammatory diseases in the elderly (e.g., Alzheimer’s and Parkinson’s disease) are known to begin at middle age and according to epidemiological data long-term use of non-steroidal anti-inflammatory drugs during this time period offers some protection against the development of these diseases (40). Our data consistently document an altered phenotype of middle-aged microglia, including high frequency, duration and integral of intracellular Ca^2 +^ signaling [this study and reference (14)]. Interestingly, the integral (area under the curve) of microglial Ca^2 +^ signals was also shown to increase (compared to WT counterparts) in ramified, located far from plaque (i.e., moderately activated) microglia in a mouse model of AD and to decrease again in strongly activated, on plaque microglia [see Figure 2 in (23)]. Based on the detailed above arguments that enhanced intracellular Ca^2 +^ signaling accompanies activation of microglia, we named the phenotype of middle-aged microglia alerted or reactive (14). Recently published single-cell RNAseq data seem to support our conclusion by showing that transiting response (i.e., partially activated) microglia is the most abundant subtype and the activated microglia build up to appr. 60% of microglia in middle-aged mice (7). The authors, however, analyzed WT and AD microglia together. Therefore, lower numbers are expected when looking only on WT microglia. Furthermore, especially at middle age we have noticed a substantial variability in the time course of Ca^2 +^ signals in female (but also male) microglia. Partially this variability might arise from the differences in the hormone status, especially different stages of the estrous cycle in female mice. Unfortunately, neither we nor the Bart De Strooper’s group (7) included the control of the hormonal status/estrous cycle in the study design. For further studies it would be, therefore, important to determine the exact hormone status of animals under study.

Microglial Ca^2 +^ signals are not only the marker for danger- or pathogen sensing by these cells but also trigger the executive functions of microglia. The latter include gene expression (41) proliferation (42), phagocytosis (43), IL-1β production, strongly associated with the activation of the NLRP3 inflammasome (44, 45), and cytoskeletal rearrangements necessary for process extension (46, 47). Thus, the analyzed here Ca^2 +^ signals likely function as an amplification element in the chain driving the change of the functional state of microglia.

### Caloric Restriction as a Tool for an Anti-aging Treatment of Microglia

Restriction of food intake is well known for its anti-inflammatory effects in man and mice [reviewed in refs. (48, 49)]. We show now that long-term caloric restriction partially prevents the aging-related changes in Ca^2 +^ signaling and potently counteracts aging-induced desynchronization in the DAMP-induced movement of microglial processes. Because under normal conditions the timeline for aging-induced changes in microglial Ca^2 +^ signaling has a bell shape, the effects of caloric restriction at the first glance seem opposite in CR3-treated middle-aged and CR6-treated old mice. In common, however, is that both treatments bring the Ca^2 +^ signaling properties of the CR-treated microglia close to those observed in the respective younger age group. Thus, the functional properties of CR3-treated middle-aged microglia look similar to that found in young control mice, whereas the properties of CR6-treated old cells resemble those of middle-aged microglia. Our data also document a pronounced CR-sensitivity of Ca^2 +^ signaling in male microglia of both (middle-aged and old) age groups and relative insensitivity to CR of the old female microglia. Notably, all changes discussed above concern the frequency and the time course of the Ca^2 +^ signals, whereas we did not observe any CR-induced changes in the fraction of active cells.

In addition, a strong effect of CR was observed for directed process motility, which is consistently dyscoordinated in old mice [this paper and reference (14)], likely because of downregulation of P2Y_12_ receptors, required for process chemotaxis, in old microglia (13). Here, the CR6-treated old microglia showed high degree of coordination of the individual processes, behaving in this respect very similar to middle-aged and even young adult mice. High coordination of individual moving processes of different cells is important for rapid clearance of cell/tissue debris as well as insulation of small blood vessel ruptures and other microdamages in the microglial environment. This also means the rapid clearance of PAMP and DAMP sources, strongly reducing the level of ambient pro-inflammatory molecules.

Having documented the potency of CR in improving the functional *in vivo* properties of aged microglia, the next critical step is to understand the underlying cellular/molecular mechanisms. One possible candidate is the ketone body β-hydroxybutyrate, concentration of which is increased by CR. In human monocytes β-hydroxybutyrate was recently shown to inhibit NLRP3 inflammasome, responsible for activation of caspase-1 and the release of the pro-inflammatory cytokines IL-1β and IL-18 (50). We consider NLRP3 inflammasome as an important mediator of described here functional changes, as it is known to be involved in both microglial Ca^2 +^ signaling as well as morphological activation of microglia (33, 36). More mechanistic insight will certainly come from single cell RNAseq and other “omics” studies comparing the transcriptomes, proteomes and metabolomes of normally aged and CR-treated mice.

Further important question concerns the applicability of the data obtained in therapeutic settings. To this end, we included a short-term CR protocol applied to old mice, to mimic the setting of the clinic. Interestingly, 6-week-long caloric restriction beginning at the age of 17–18 months mimicked the strongest effects of long term caloric restriction concerning both the changes in microglial Ca^2 +^ signaling and in the process motility. These data show that at least in mice caloric restriction can be beneficial even when started at old age and does have under these conditions substantial anti-aging and anti-inflammatory properties. Because the mentioned above NLRP3 inflammasome is overtly activated in AD patients as well as in mouse models of the disease and its inhibition was identified as a new therapeutic target for treating both neuronal and microglial dysfunction in AD (33, 51), we hypothesize that caloric restriction has a potential to alleviate the consequences of amyloid ß deposition in mice and humans. Interestingly, a small-molecule inhibitor of NLRP3 was recently shown to attenuate the severity of experimental autoimmune encephalomyelitis, a disease model of multiple sclerosis (52). Thus, CR-mediated inhibition of NLRP3 might also have potential for treating autoinflammatory and autoimmune disorders.

Together, our data show that aging microglia changes its phenotype not once but at least twice and “immune alerted” or “primed” phenotype is inherent to middle-aged rather than old microglia. Despite the common aging-related changes described above, at any age the fine tuning of microglial Ca^2 +^ signaling is controlled by the animal’s sex. Finally, our data identify the CR as a potent means for both prevention (CR3, CR6 protocols) and reversal (CR18 protocol) of aging-related functional changes in microglia.

## Data Availability Statement

The datasets generated in this study are available from the corresponding author upon a reasonable request.

## Ethics Statement

All experimental procedures followed the Directive 2010/63/EU of the European Parliament and the Council of the European Union, were carried out in accordance with institutional animal welfare guidelines. The animal study was reviewed and approved by Regierungspräsidium Tübingen.

## Author Contributions

OG conceived the study. NF and MO designed the experiments. MO performed the experiments and analyzed the data. KF and NM analyzed the RNAseq data. NF, MO, and OG wrote the manuscript. All authors commented on the manuscript.

## Conflict of Interest

The authors declare that the research was conducted in the absence of any commercial or financial relationships that could be construed as a potential conflict of interest.

## Footnotes

1 http://fiji.sc/Fiji

2 https://imagej.net/MTrackJ
