# Supplementary material for: Effect of Caloric Restriction on the in vivo Functional Properties of Aging Microglia
Source: Front Immunol. 2020 Apr 28;11:750. doi: 10.3389/fimmu.2020.00750 (PMC7198715; doi:10.3389/fimmu.2020.00750)
Supplement: Supplementary file 1 [file Data_Sheet_1.pdf]

Supplementary material

Effect of caloric restriction on the *in vivo* functional properties of aging microglia

Maria Olmedillas del Moral, Nicole Fröhlich, Katherine Figarella, Nima Mojtahedi, Olga Garaschuk*

Department of Neurophysiology, Institute of Physiology, Eberhard Karls University Tübingen, Tübingen, Germany

*** Correspondence:**
Olga Garaschuk
olga.garaschuk@uni-tuebingen.de

Supplementary Material

Supplementary Figure 1. Normalized gene expression values of transcripts differentially expressed in 3-month-old WT male and female mice. The genes of interest were grouped according to different GO annotations indicated on the top of the respective panel. Box plots illustrate **(A)** GO annotations associated with inflammation (13 genes), **(B)** differentially expressed genes encoding K^+ channels (26 genes), **(C)** GO annotations associated with regulation of the cytosolic Ca^{2+} concentration (24 genes). The latter group was subdivided into GO terms related to **(D)** release of sequestered Ca^{2+} into the cytosol (13 genes), **(E)** Ca^{2+}-mediated signaling using intracellular Ca^{2+} source (5 genes), and **(F)** positive regulation of cytosolic Ca^{2+} ion concentration (8 genes). Statistical differences were determined using Mann-Whitney test (*$p < 0.05$, ***$p < 0.001$).

Supplementary Figure 2. Effect of caloric restriction on spontaneous Ca^{2+} signals in middle-aged male mice. Box-and-whisker plots illustrating the median (per cell) frequency **(A)**, amplitude **(B)**, T-half **(C)** and AUC **(D)** of spontaneous Ca^{2+} transients in microglia from CR3 male mice (n = 9 cells, 5 mice). Statistical differences were determined using Mann-Whitney test (*$p < 0.05$, **$p < 0.01$).

Supplementary Figure 3. No sex differences in chemotactic properties of microglia. Box-and-whisker plot illustrating the effect of the age and sex on the median (per mouse) Spearman's correlation coefficients between the initial distance of microglial processes to the tip of the ATP-containing pipette and the mean process velocity (n = 4 males, 7 females for 2-4 months old mice; 7 males, 4 females for 9-11 months old mice and 9 males, 4 females for 18-21 month old mice). Data are pooled from WT and $CX_3CR1^{GFP/+}$ mice.
