# Supplementary material for: Effect of Caloric Restriction on the in vivo Functional Properties of Aging Microglia
Source: Front Immunol. 2020 Apr 28;11:750. doi: 10.3389/fimmu.2020.00750 (PMC7198715; doi:10.3389/fimmu.2020.00750)
Supplement: Supplementary file 2 [file Table_1.pdf]

Table S1. GO annotations and genes included in each condition

Group	Gene symbol	Gene name
**Inflammation**		
GO:0002526, acute inflammatory response; GO:0001774, microglial cell activation; GO:0050729, positive regulation of inflammatory response	Il1b	Interleukine 1 beta
	Spn	Sialophorin
	Ccl5	C-C Motif Chemokine Ligand 5
	DNase1|3	Deoxyribonuclease gamma
	Ccr7	C-C chemokine receptor type 7
	F8	Coagulation factor VIII
	Il1rn	Interlekin 1 receptor antagonist
	Saa3	Serum amyloid A 3
	Serpinf2	Serpin family F member 2
	Sigirr	Single immunoglobulin domain-containing IL1R-related protein
	Clu	Clusterin
	Ager	Advanced glycosylation end product-specific receptor
	Mapt	Microtubule-associated protein tau
**Potassium channels**		
	Kcna2	Potassium Voltage-Gated Channel Subfamily A Member 2
	Kcna3	Potassium Voltage-Gated Channel Subfamily A Member 3
	Kcnb2	Potassium Voltage-Gated Channel Subfamily B Member 2
	Kcnd2	Potassium Voltage-Gated Channel Subfamily D Member 2
	Kcnd3	Potassium Voltage-Gated Channel Subfamily D Member 3
	Kcnc2	Potassium Voltage-Gated Channel Subfamily C Member 2
	Kcnh3	Potassium Voltage-Gated Channel Subfamily H Member 3
	Kcnh1	Potassium Voltage-Gated Channel Subfamily H Member 1
	Kcnh4	Potassium Voltage-Gated Channel Subfamily H Member 4
	Kcnh7	Potassium Voltage-Gated Channel Subfamily H Member 7
	Kcnj3	Potassium Inwardly Rectifying Channel Subfamily J Member 3
	Kcnj10	Potassium Inwardly Rectifying Channel Subfamily J Member 10
	Kcnj15	Potassium Inwardly Rectifying Channel Subfamily J Member 15
	Kcnk1	Potassium Two Pore Domain Channel Subfamily K Member 1
	Kcnk2	Potassium Two Pore Domain Channel Subfamily K Member 2
	Kcnk7	Potassium Two Pore Domain Channel Subfamily K Member 7
	Kcnn2	Potassium Calcium-Activated Channel Subfamily N Member 2
	Kcnn3	Potassium Calcium-Activated Channel Subfamily N Member 3
	Kcnrg	Potassium Channel Regulator
	Kcnv1	Potassium Voltage-Gated Channel Modifier Subfamily V Member 1
	Kcnip2	Potassium Voltage-Gated Channel Interacting Protein 2
	Kcnmb1	Potassium Calcium-Activated Channel Subfamily M Regulatory Beta Subunit 1
	Kcnmb4	Potassium Calcium-Activated Channel Subfamily M Regulatory Beta Subunit 4
	Kcnq2	Potassium Voltage-Gated Channel Subfamily Q Member 2

	Kcnq3	Potassium Voltage-Gated Channel Subfamily Q Member 3
	Kcnt1	Potassium Sodium-Activated Channel Subfamily T Member 1
**Calcium related pathways**		
GO:0051209, release of sequestered calcium into cytosol	Htr2a	5-Hydroxytryptamine (serotonin) receptor 2a
	Htr2b	5-Hydroxytryptamine (serotonin) receptor 2b
	Ryr1	Ryanodine receptor 1
	Ryr2	Ryanodine receptor 2
	Ryr3	Ryanodine receptor 3
	Plcd4	Phospholipase C, delta 4
	Plcb1	Phospholipase C, beta 1
	Plcd3	Phospholipase C, delta 3
	Plch2	Phospholipase C, eta 2
	Plch1	Phospholipase C, eta 1
	Lck	Lymphocyte protein tyrosine kinase
	Xcr1	Chemokine (C motif) receptor 1
	Bbc3	BCL2 binding component 3
GO:0035584, calcium-mediated signaling using intracellular calcium source	Kdr	kinase insert domain protein receptor
	Homer2	homer scaffolding protein 2
	Ryr2	Ryanodine receptor 2
	Selp	Selectin, platelet
	Ntrk2	Neurotrophic tyrosine kinase, receptor, type 2
GO:0007204, positive regulation of cytosolic calcium ion concentration	Cacnb4	Calcium channel, voltage-dependent, L type, beta 4 subunit
	Ccl28	Chemokine (C-C motif) ligand 28
	Cav1	Caveolin 1
	Synpo	Synaptopodin
	Gper1	G protein-coupled estrogen receptor 1
	Bak1	BCL2 Antagonist/Killer 1
	Trpv6	Transient receptor potential cation channel, subfamily V, member 6
	Xcr1	Chemokine (C motif) receptor 1
