# Supplementary material for: Effect of Caloric Restriction on the in vivo Functional Properties of Aging Microglia
Source: Front Immunol. 2020 Apr 28;11:750. doi: 10.3389/fimmu.2020.00750 (PMC7198715; doi:10.3389/fimmu.2020.00750)

Figure S1. Normalized gene expression values of transcripts differentially expressed in 3-month-old WT male and female mice

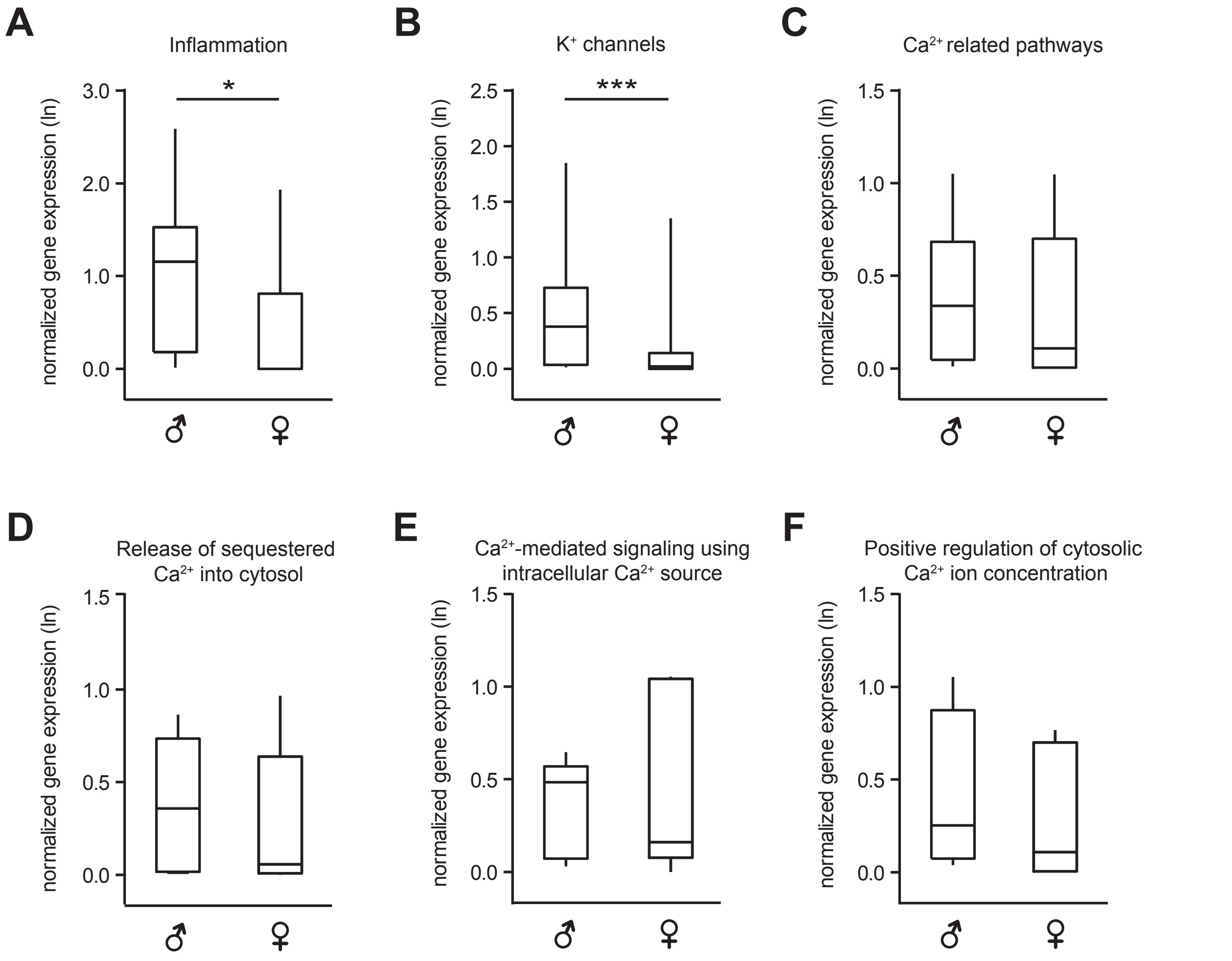

Supplement: Supplementary file 3 [file Image_1.pdf]
