# Supplementary figures and images for: Effect of Caloric Restriction on the in vivo Functional Properties of Aging Microglia
Source: Front Immunol. 2020 Apr 28;11:750. doi: 10.3389/fimmu.2020.00750 (PMC7198715; doi:10.3389/fimmu.2020.00750)

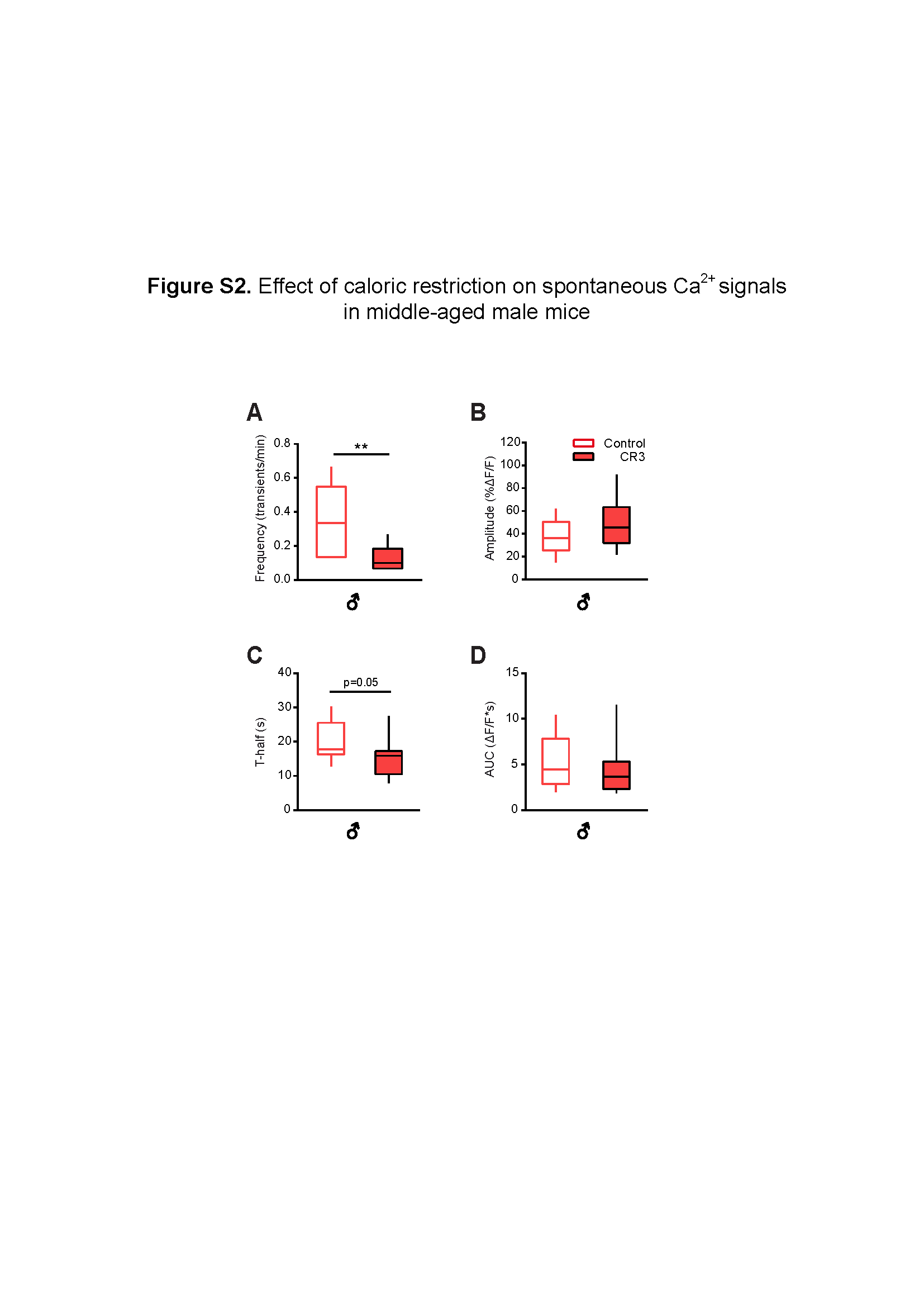

Supplement: Supplementary file 4 [file Image_2.TIF]

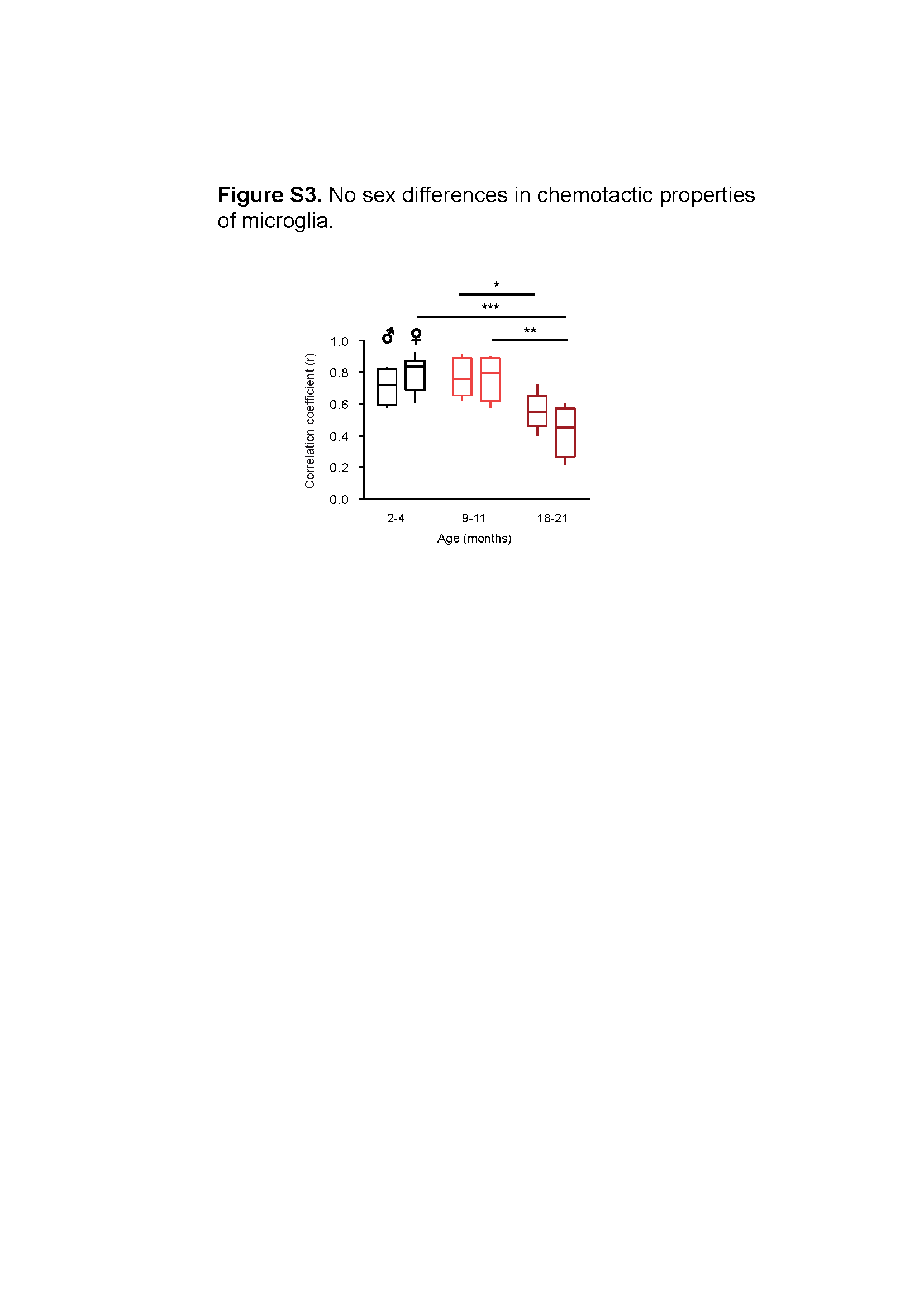

Supplement: Supplementary file 5 [file Image_3.TIFF]
